# Corrosion Behavior and Mechanism of Basalt Fibers in Sodium Hydroxide Solution

**DOI:** 10.3390/ma11081381

**Published:** 2018-08-08

**Authors:** Chunhong Tang, Hao Jiang, Xu Zhang, Guangyao Li, Junjia Cui

**Affiliations:** 1 State Key Laboratory of Advanced Design and Manufacturing for Vehicle Body, Hunan University, Changsha 410082, China; chun@hnu.edu.cn (C.T.); haojiang@hnu.edu.cn (H.J.); gyli@hnu.edu.cn (G.L.); 2 Joint Center for Intelligent New Energy Vehicle, Shanghai 200092, China; 3 College of Automotive and Mechanical Engineering, Changsha University of Science and Technology, Changsha 410004, China; ddzhangxu@126.com

**Keywords:** basalt fiber, alkaline corrosion, corrosion behavior, mechanical property

## Abstract

In this paper, the corrosion mechanism and tensile properties of basalt fibers in sodium hydroxide (NaOH) solution with various concentrations and temperatures were studied. The hydroxyl ions disrupt the –Si–O–Si– and –Si–O–Al– bonds leading to the formation of insoluble hydroxides. With the continuation of the hydration reaction, a hydration layer (corrosion shell) with high content of calcium, iron, manganese and titanium ions was formed on the fiber surface. The corrosion shell enabled an increase in the strength and elongation at break of basalt fibers, significantly. Results showed that the tensile strength of fibers was strongly dependent on temperature and concentration. After the basalt fibers were immersed in 1 mol/L NaOH solution at 50 °C for 1 h, 3 h, 6 h, 1 day and 3 days, their retention ratios of strength were 67.6%, 57.8%, 52.5%, 49.0%, 58.2%, respectively. Higher temperature accelerated the corrosion rate of basalt fibers, shortened the formation time of the corrosion shell and increased mass loss. From 25 to 70 °C, the mass loss of fibers increased from 2.4% to 33.8% for fibers immersed in 1 mol/L NaOH for 3 days. The experimental results from quantitative x-ray fluorescence (XRF) showed that the mass loss of basalt fibers was mainly due to the leaching of silicon, aluminum and potassium ions.

## 1. Introduction

Composite materials reinforced with fibers have been widely used in machinery, automobile, and other industries [1,2,3,4]. The basalt fiber, produced from basalt stone, is a kind of a high-tech fiber not harmful to human beings [5]. The application temperature range of basalt fibers is –200 to +700 °C. In contrast, the application temperature range of glass fiber is only −60 to +450 °C [6]. In addition, basalt fibers have similar or better mechanical property—improved thermal and chemical stability—than that of E-glass fibers [7,8,9]. Due to advantages such as higher application temperature, harmlessness to human beings and excellent resistance to alkaline and acid attack, basalt fibers are increasingly being used to replace glass fiber as a reinforcement material in polymer, concrete and metallic matrices [5,10,11,12]. However, chemical corrosion is one of the most challenging issues faced during the service process of composites [13]. Take basalt fibers-reinforced concrete as an example—they are usually corroded by the high alkalinity of the cement matrix [14,15,16]. There has also been an increased interest in using basalt fibers to manufacture filter materials in recent years [17]. Certain types of filter gas can destroy basalt fibers under high temperature and high alkali. Therefore, understanding the chemical resistance of basalt fibers in an alkaline environment is significantly important.

The present investigation on corrosion of basalt fibers in an alkaline environment mainly focuses on two aspects: chemical composition analysis [18,19] and tensile properties of degraded basalt fibers [20,21]. The long-term chemical stability of inorganic fibers could hardly be explicitly measured [22], and thus most researchers performed accelerated-aging tests [20,23,24].

With regard to chemical composition analysis of degraded basalt fibers, Ramachandran, et al. [25] investigated the alkali resistance of basalt fibers in sodium hydroxide (NaOH) solution, and found that the presence of TiO_2_, MnO, Fe_2_O_3_ and Al_2_O_3_ could slow alkaline corrosion. Li. et al. [26] evaluated the corrosion behavior of basalt fibers under different alkaline solution concentrations and at room temperature. It was concluded that the element contents of aluminum, calcium and iron of the fiber surface were not sensitive to the concentration. The chemical resistance of zirconia-coated basalt fibers in alkali solution was investigated by Rybin, et al. [27]. It was found that the dense zirconia coating was more effective than the porous coating in protecting the fiber against corrosion.

In addition, as for tensile properties of degraded basalt fibers, Wu, et al. [20] investigated the degradation of basalt fibers subjected to 25 and 55 °C alkaline solution. The results showed that tensile strength respectively decreased to 33.8%, 18.0%, 10.2% and 5.8% at a temperature of 25 °C after ageing for 7, 18, 34 and 66 days. When the temperature rose to 55 °C, the strength decreased to 33.0%, 17.7%, 10.6% and 0, respectively. This meant that the higher temperature had no significant effect on the degradation of tensile strength, except when ageing for 66 days. Furthermore, Ying and Zhou [21] evaluated the tensile strength of basalt fibers in 1 mol/L NaOH solution with temperature of 100 °C. They found that the tensile strength first decreased and then rose with the increase of corrosion time.

The variation trends of the tensile strength of degraded basalt fibers were of two main types: (a) Continually decreasing with increase of corrosion time [20,22] and (b) decreasing first and then growing with increase of corrosion time [21].

It is widely accepted that the strength degradation was caused by the mobility of hydroxyl ions [26,28]. When the concentration was over a certain value, the mobility of hydroxyl ions decreased. This would result in the reduced corrosion of fibers. However, this reason could not explain why the tensile strength decreased first and then increased with increasing corrosion time. Moreover, the effect of temperature, solution concentration and corrosion time on the tensile properties of degraded basalt fibers immersed in strong alkali solution, and the chemical corrosion of basalt fibers in NaOH solution with different temperatures and concentrations has not been systematically studied so far.

In general, the corrosion solutions used for simulating alkaline environments were: NaOH solution, cement solution, calcium hydroxide (Ca(OH)_2_) solution and solutions containing a mixture of NaOH, Ca(OH)_2_ and potassium hydroxide (KOH) [23,27,29,30,31]. Cement and calcium hydroxide are used to simulate the environment of building materials. The mixed solution is usually used to simulate an alkaline environment with a variety of ions. In order to simulate the high alkali environment that the basalt fibers may encounter during the application of filter material, the NaOH solution was selected as the etchant in this study.

Therefore, the aim of the present work is to investigate the tensile properties of degraded continuous basalt fibers after immersing in NaOH solution with different concentrations and at different temperatures. Firstly, the effects of temperature and solution concentration on the mass loss and mechanical performance of basalt fibers were investigated. The surface morphologies of basalt fibers in NaOH solution with a series of temperatures and concentrations were examined by scanning electron microscopy (SEM). Finally, energy dispersive spectrometry (EDS) was conducted to identify the element changes of the marked points on the surface of basalt fibers.

## 2. Materials and Methods

### 2.1. Experimental Materials

**Figure 1:**
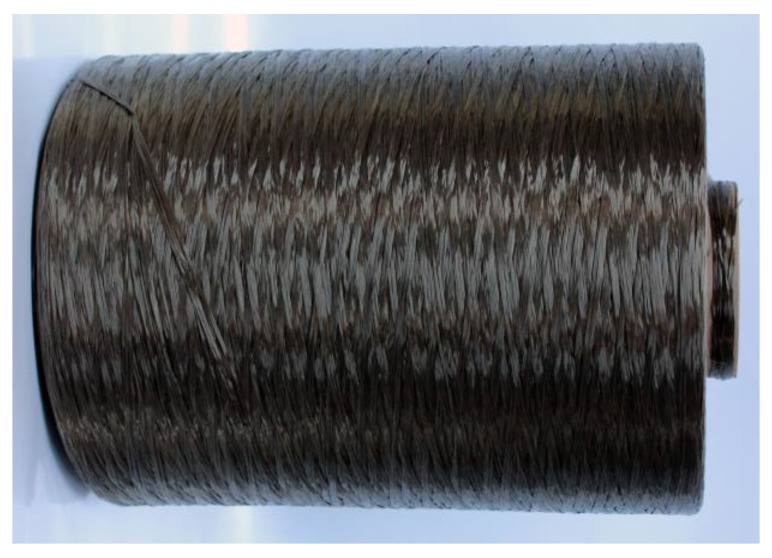
Continuous untwisted basalt fibers.

Continuous untwisted basalt fibers (see Figure 1) were used in this study. In order to remove the sizing emulsifier used in the spinning process, basalt fibers were first soaked in acetone for a long period and then washed with distilled water. Finally, they were dried for 40 min at 105 °C. The average diameter of a single desized fiber was about 16 μm. The chemical compositions of desized basalt fibers were examined by quantitative x-ray fluorescence (XRF) analysis; the main chemical components are given in Table 1. The most important components of basalt fibers were SiO_2_, Fe_2_O_3_, Al_2_O_3_, CaO and MgO. The basic mechanical properties of the desized basalt fibers are shown in Table 2.

### 2.2. Fiber Treatment

To investigate the effects of temperatures and concentrations on the corrosion behavior of basalt fibers, the fibers were immersed in NaOH solution at temperatures of 25, 50 and 70 °C and the concentrations of 1, 2, 3 and 4 mol/L, respectively. The mass ratio of fiber/NaOH solution for strength test and mass loss test was about 1/250 and 1/450, respectively. The fibers were placed in a special plastic bottle containing NaOH solution and then sealed with parafilm. The fibers were then taken out from the plastic bottle and washed with distilled water after immersing for a defined corrosion period. Finally, all specimens were dried in a vacuum oven at 105 °C for 40 min.

### 2.3. Measurement and Characterization

The tensile properties of the basalt fiber filaments were examined using a fiber tensile tester with a gauge length of 20 mm according to a modified ISO 11566 test method. Tensile load was applied on the filaments under a loading rate of 2 mm/min. The retention ratio of tensile properties was calculated according to Equation (1):

(1) Retentio n ratio (%) = Degraded tensile property Original tensile property×100%

where the tensile properties include tensile strength and elongation at break.

The mass loss involved weighing the fibers after various treatment periods in the NaOH solution. In order to prevent the mass of the basalt fibers from being affected by the drying process, each of the weighed samples was used only for one specific treatment period. The mass of basalt fibers before and after corrosion were weighed using an electronic analytical balance and the mass losses of each specimens were estimated according to Equation (2):

(2) $$Mass\;loss\;(\%)\;=\;\frac{M_{0}-M_{1}\;}{M_{0}}\times 100\%$$

where *M*_0_ and *M*_1_ were the mass of fibers before and after the treatments, respectively.

The surface morphologies of the basalt fibers before and after corrosion were examined by a FEI Quanta 200 scanning electron microscope (SEM) (FEI: Prague, Czech Republic). The element changes of the marked points on the surface of basalt fibers were examined with an EDAX Genesis 2000 energy dispersive spectrometer (EDS) (EDAX: Mahwah, NJ, USA).

## 3. Results and Discussion

### 3.1. Mass Loss

**Figure 2:**
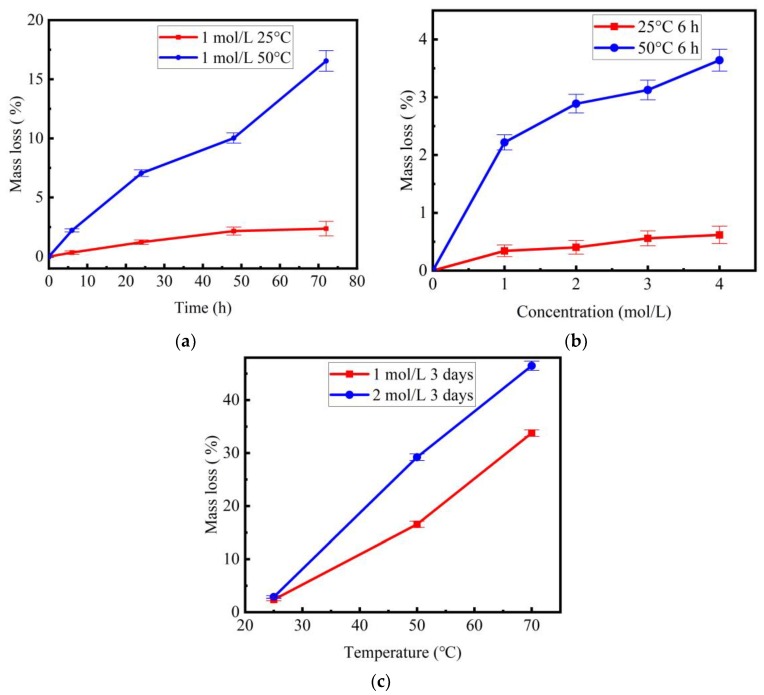
Mass loss ratios of basalt fibers after NaOH solution treatment: (**a**) mass loss ratios vs. corrosion time; (**b**) mass loss ratios vs. concentration with 6 h immersion; (**c**) mass loss ratio vs. temperature with 3 days′ immersion.

The mass loss ratios of the degraded basalt fibers versus corrosion time, concentration and temperature are illustrated in Figure 2a–c, respectively. As shown in Figure 2a, it was observed that the mass loss ratios of basalt fibers were quite stable as immersed time increased at temperature of 25 °C. However, there was a significant weight drop at temperature of 50 °C. More specifically, when the temperature was 25 °C, the mass loss ratios of fibers with corrosion time of 6 h, 1 day, 2 days and 3 days were 0.3%, 1.2%, 2.2% and 2.4%, respectively. When the temperature was 50 °C, the mass loss ratios of fibers with corrosion time of 6 h, 1 day, 2 days and 3 days were 2.2%, 7.1%, 10.0%, and 16.6%, respectively. From Figure 2b, it can be seen that the mass loss of fibers at 50 °C was much higher than that at 25 °C. Figure 2c shows that the mass loss value of fibers with 3 days’ treatment drastically increased when the temperature increased from 25 °C to 70 °C. For example, at a concentration of 1 mol/L and corrosion time of 3 days, the mass loss ratios of basalt fibers at 25, 50 and 70 °C were 2.4%, 16.6% and 33.8%, respectively. It could be concluded that the mass loss of the basalt fibers was significantly affected by temperature, corrosion time and solution concentration. Mass loss, especially, increased with the increasing of temperature. This further demonstrated that higher temperature accelerated the corrosion of basalt fibers in alkaline solution.

The basalt fibers before and after corrosion were examined by quantitative XRF analysis; the main chemical compositions of desized and degraded fibers are given in Table 3. In comparison with desized basalt fibers, the content of SiO_2_, Al_2_O_3_, and K_2_O decreased by 13.1%, 7.0% and 1.1%, respectively. This phenomenon was mainly caused by the leaching of silicon, aluminum and potassium ions.

### 3.2. Tensile Behavior of Basalt Fibers

**Figure 3:**
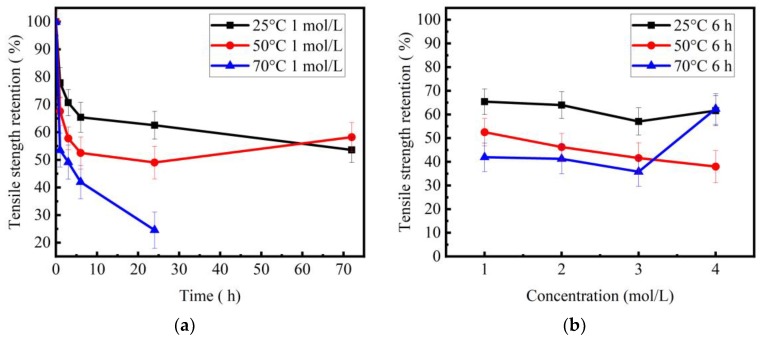
Tensile strength retention ratios of basalt fibers after NaOH solution treatment: (**a**) tensile strength retention ratios vs. corrosion time; (**b**) tensile strength retention ratios vs. concentration with 6 hr immersion.

The retention ratios of tensile strength for basalt fibers immersed in NaOH solutions versus corrosion time and concentration are shown in Figure 3a,b, respectively. On the whole, the tensile strength of basalt fibers at 70 °C degraded more seriously than at 25 °C. The temperature had a great influence on the variation trend of tensile strength with increase of corrosion time. For example, when the temperature was 25 °C, the tensile strength of basalt fibers decreased as corrosion time increased. After 1 h, 3 h, 6 h, 1 day and 3 days, its retention ratios were 77.9%, 70.7%, 65.4%, 62.5%, 53.6%, respectively. When the temperature rose to 50 °C, tensile strength first decreased and then increased with increase of corrosion time. After 1 h, 3 h, 6 h, 1 day and 3 days, its retention ratios were 67.6%, 57.8%, 52.5%, 49.0%, 58.2%, respectively. When the temperature was 70 °C, the tensile strength of degraded basalt fibers decreased as corrosion time increased. After 1, 3, 6 and 24 h, its retention ratios were 53.5%, 49.2%, 42.0% and 24.6%, respectively.

Figure 3b shows the tensile strength of basalt fibers versus solution concentration with 6 h treatment. Specifically, when the temperature was 25 °C, the tensile strength first decreased and then increased with increase of concentration. Its retention ratios were 65.4%, 64.0%, 57.1% and 61.6% when the concentrations were 1, 2, 3 and 4 mol/L, respectively. The lowermost tensile strength appeared at the concentration range of 2–4 mol/L, in this study. When the temperature rose to 50 °C, the tensile strength of basalt fibers decreased with the increase of concentration. Its retention ratios were 52.5%, 46.2%, 41.6% and 38.0% when the concentrations were 1, 2, 3 and 4 mol/L, respectively. When the temperature was 70 °C, the tensile strength of basalt fibers first decreased and then increased as the concentration increased from 1 to 4 mol/L. Its retention ratios were 42.0%, 41.3%, 35.8% and 62.4% when the concentrations were 1, 2, 3 and 4 mol/L, respectively. The lowest tensile strength occurred in 3 mol/L. Moreover, its retention value dramatically increased to 62.4% at a concentration of 4 mol/L.

**Figure 4:**
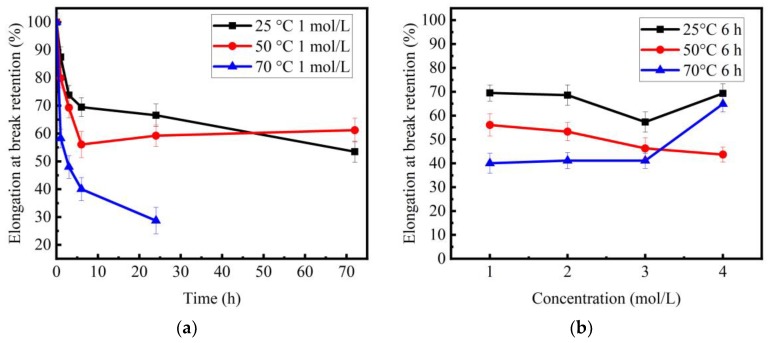
Elongation at break retention ratios of basalt fibers after NaOH solution treatment: (**a**) elongation at break retention ratios vs. corrosion time; (**b**) elongation at break retention ratios vs. concentration with 6 h immersion.

Figure 4 shows the retention ratios of elongation of basalt fibers with NaOH treatment versus corrosion time and concentration, respectively. It was noted that elongation at break and tensile strength have the same variation tendency. It is worth noting that the lowest tensile strength occurred at the corrosion time of one day (Figure 3a) when the temperature was 50 °C. However, the lowest elongation occurred at the 6 h corrosion time (Figure 4a).

### 3.3. SEM Image Analysis

**Figure 5:**
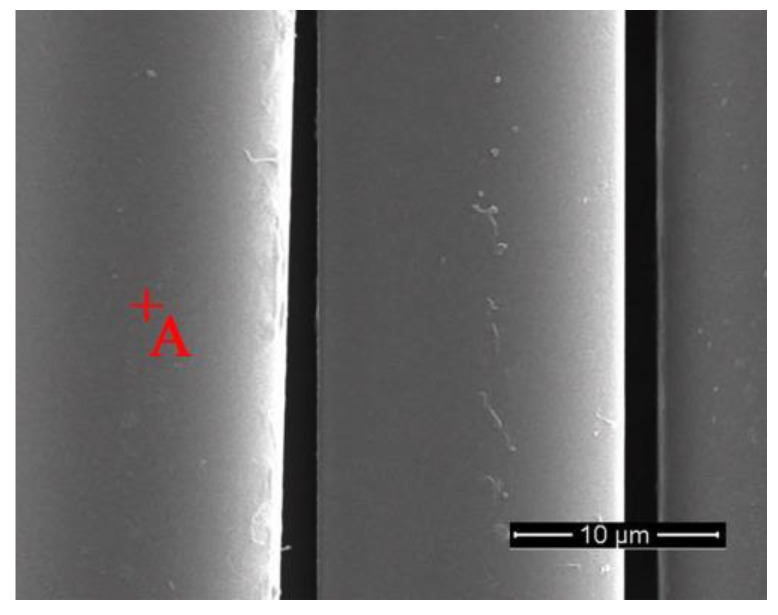
SEM images of desized basalt fibers.

Desized basalt fibers had a fairly smooth surface (Figure 5), and no visible pores and micro-cracks were observed.

**Figure 6:**
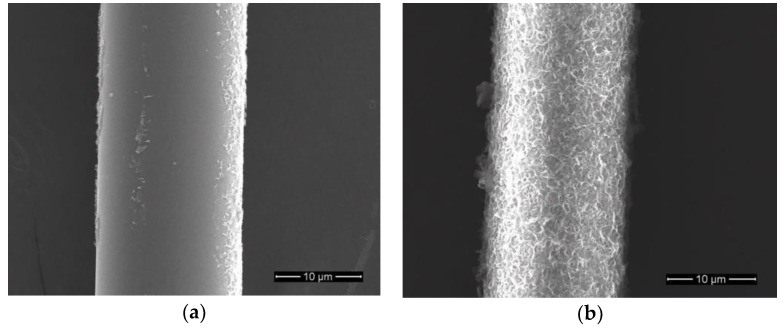
SEM images of basalt fibers immersed in 1 mol/L NaOH solution for 3 days: (**a**) 25 °C; (**b**) 50 °C.

The SEM images of basalt fibers immersed in 1 mol/L NaOH solution at temperatures of 25 °C and 50 °C for 3 days are shown in Figure 6. Compared with the surface condition of desized fiber, a flaky corrosion layer appeared in some areas of the fiber surface, but no visible cracks and pores were observed when the temperature was 25 °C. However, a complete corrosion shell covering the entire fiber surface was observed when the temperature rose to 50 °C. It can be concluded that the morphology of the fiber surface was significantly affected by temperature and the higher temperature accelerated formation of the corrosion shell.

**Figure 7:**
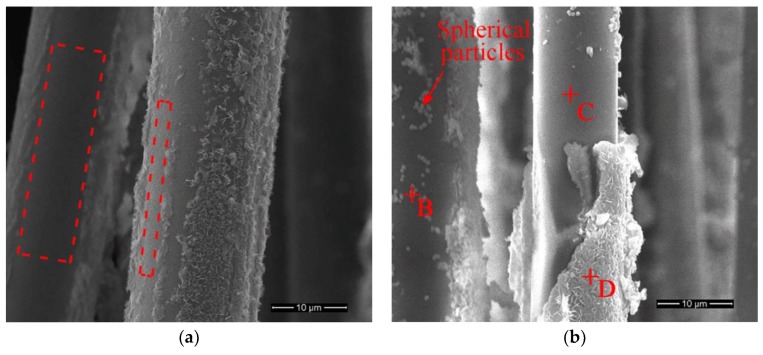
SEM images of basalt fibers treated in NaOH solution at 70 °C for 6 h: (**a**) 1 mol/L; (**b**) 4 mol/L.

Figure 7 shows the basalt fibers immersed in different concentrations of NaOH solution at 70 °C for 6 hours. At a concentration of 1 mol/L, several flaky corrosion layers appeared on the fiber surface, but did not completely cover it. The areas marked with dotted lines were quite smooth and had straight borders. Corrosion layers formed on the surface and slightly adhered to the fiber core after corrosion (due to contact of the fibers before corrosion). During the drying and measuring process, the relative position between fibers changed, which resulted in exfoliation of the corrosion layer. Therefore, smooth areas with straight borders were observed. Numerous spherical particles formed on the surface of basalt fiber at a concentration of 4 mol/L. In addition, a corrosion shell, partially peeled off, was observed and the corrosion shell peeled off created a smooth “fresh” surface. It could be concluded that the morphology of the fiber surface was significantly affected by solution concentration. At a higher temperature, the higher solution concentration accelerated the formation of the corrosion shell.

**Figure 8:**
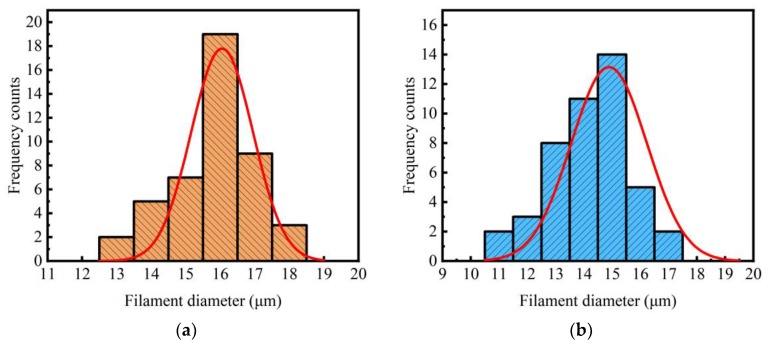
Distributions of the filament diameters: (**a**) initial state; (**b**) immersed in 4 mol/L NaOH solution at 70 °C for 6 h.

The peeled off corrosion shells were analyzed by the distribution law of basalt fiber filament diameters (see Figure 8). The diameters were determined on each fiber filament before tensile tests. It could be seen that the vast majority of diameters of basalt fibers were 16 μm in the initial state. However, after several hours of immersion, the diameters of most basalt fibers were about 14 and 15 μm.

**Figure 9:**
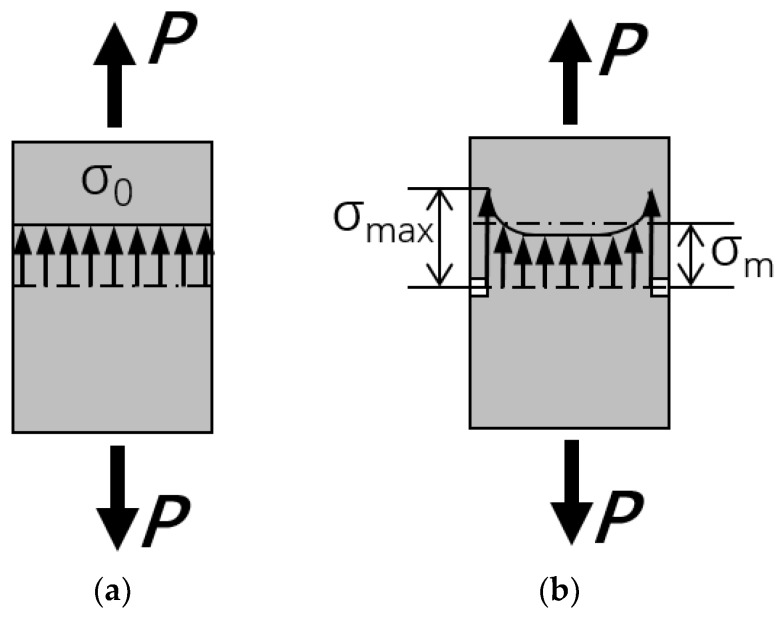
Schematic diagrams of stress distribution: (**a**) non stress concentration; (**b**) stress concentration.

In order to explain the reason for increase of tensile strength when corrosion shell is formed on the fiber surface, it was important to introduce the concept of stress concentration. Stress concentration refers to the phenomenon where the maximum stress value of a local region of a structure or component is higher than the average stress value. Figure 9 shows the schematic diagrams of stress distribution, with and without stress concentration. When a force *P* is applied to the non-defective fiber (Figure 9a), the stress distribution of the fiber is almost uniform. Fiber fails at σ_0_ = σ*_b_*, where σ*_b_* represents the ultimate tensile stress of material. When the force *P* is applied to the defective fiber (Figure 9b), the stress distribution of the fiber is not uniform, and the stress near the defect is much higher than the average stress. Fiber fails at σ_max_ = σ*_b_*. In this case, the tensile strength we measured (σ*_m_*) is less than σ*_b_*. The higher the degree of stress concentration, the larger σ_max_ and the smaller σ*_m_*.

Before the formation of the corrosion shell, the degree of stress concentration increased, and the tensile strength of the fiber decreased as the corrosion time increased. This reduced the degree of stress concentration when the corrosion shell was formed (see the next section for a detailed analysis), resulting in an increase in tensile strength. In addition, on comparing the fiber with corrosion shell (treated with 1 mol/L NaOH for 3 days at 25 °C) and without corrosion shell (treated with 1 mol/L NaOH for 3 days at 50 °C), it could be seen that the decrease in tensile strength caused by stress concentration was greater than that caused by the corrosion shell.

Assuming that the corrosion shell is unable to bear the pulling force, the effect of the corrosion shell on tensile strength retention, namely *T_s_*, could be estimated by the following formula:

(3) $$T_{s}\;=\;\frac{\pi {\left(\frac{d_{0}}{2}-h\right)}^{2}\;}{\pi (\frac{d_{0}}{2}){}^{2}}\times 100\%=\;{\left(1-\frac{2h}{d_{0}}\right)}^{2}\times 100\%\;$$

where *d*_0_ is the diameter of the single filament before corrosion, and *h* is the thickness of corrosion shell.

From the SEM images, the diameters of filaments before and after corrosion were obtained as *d*_0_ = 13.1–18.4 μm*. h =* 1.2–1.5 μm, respectively. Therefore, the value of *T_s_* was estimated as 59.4–75.6%.

**Figure 10:**
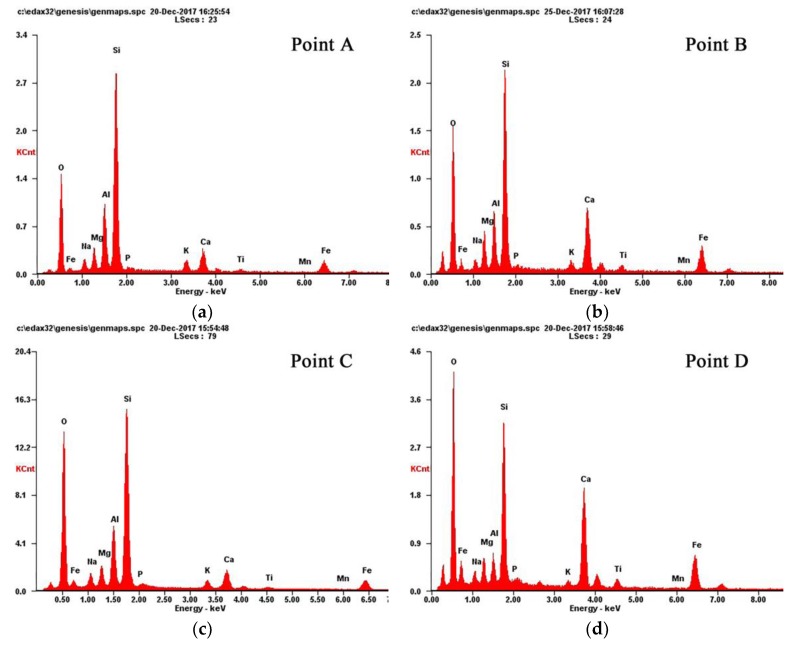
EDS results of basalt fibers: (**a**) Point A; (**b**) Point B; (**c**) Point C and (**d**) Point D.

Figure 10 shows the EDS spectra of the marked points of Figure 5 and Figure 7; the microanalysis results are given in Table 4. Compared with Point A (desized fiber), the element content of calcium, iron, magnesium and titanium on point B increased significantly. This meant that these metal ions were deposited on the surface of basalt fibers. It was also found that the surface underneath the corrosion shell (Point C) had similar element content as the initial state (desized fiber). The corrosion shell (Point D) indicated that the content of calcium, iron, manganese and titanium ions increased. However, the element content of aluminum, silicon and potassium drastically decreased. These EDS results were consistent with the results of the XRF analysis (Table 3), which further confirmed that a large number of aluminum, silicon and potassium ions leached out from the basalt fiber surface. This was the main reason for the mass loss of basalt fibers.

## 4. Further Discussions

### 4.1. Corrosion Process of Basalt Fibers in NaOH Solution

**Figure 11:**
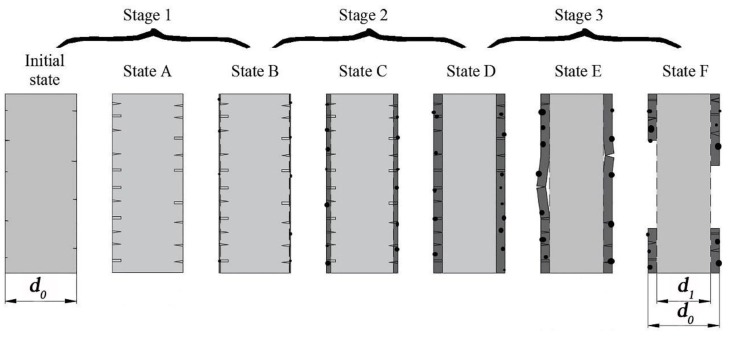
Schematic drawing of the corrosion process of basalt fibers in NaOH solution.

Studies have been conducted on the chemical stability of glass or glass fibers in alkaline or aqueous solution [32,33]. As basalt fibers had similar chemical compositions to glass fibers, there were similarities between the two. Based on the above results and analysis, the schematic corrosion process of basalt fibers in NaOH solution is described in Figure 11. The corrosion process could be broadly divided into three corrosion stages: the silicate dissolution stage (Stage 1), the formation and growing of the corrosion shell (Stage 2) and the corrosion shell peeled off (Stage 3).

In the initial state, the surface of the basalt fibers seemed fairly smooth. Although the surface of basalt fibers were assumed to include some defects (micro-cracks and pores), they were hardly visible because the sizes were too small. In Stage 1, the hydroxyl ions of the NaOH solution disrupt the –Si–O–Si– and –Si–O–Al– bonds similar to the glass fiber in an alkaline environment [30,33]. The aluminosilicate networks began to dissolve through the micro-cracks and pores, leading to small pores and micro-cracks that extend to the core of the fiber. As a consequence, the degree of stress concentration increased, causing the tensile strength to decrease quickly. In addition, the hydration reaction accompanied the basalt fiber dissolution, and insoluble hydroxides formed by calcium and iron covered the fiber surface. A thin hydrated layer (corrosion shell), which covered the complete filament surface, was formed (State B) with continuation of the hydration reaction. The corrosion shell acted as a protective layer and slowed down the dissolution rate of fibers.

When passing State B, the corrosion process progressed to Stage 2. As corrosion time increased, the growth of the corrosion shell gradually slowed down the diffusion rate of hydroxyl ions into the fiber core and thus reduced the dissolution rate of silicate. One end of the pores and micro-cracks were in the corrosion shell and the other end in the fiber core (see State C). The tensile strength of basalt fibers was affected by the corrosion shell, the pores and micro-cracks. The basalt fibers had very low tensile strength in this corrosion state.

In State D, the corrosion shell was the thickest. Almost all the pores and micro-cracks were located in the corrosion shell. Due to the lower tensile strength of the corrosion shell and with the corrosion shell weakly bonded to the fiber core, the degree of stress concentration was reduced effectively. The tensile strength of the basalt fibers was mainly affected by the thickness of corrosion shell in this corrosion state. Relative to the larger fiber diameter, the thickness of corrosion shell had less effect on the decrease of tensile strength than the effect of stress concentration on the decrease of tensile strength. Therefore, the tensile strength of basalt fibers increased at State D when compared to State C.

Water molecules penetrated the glass network [34,35], leading to increasing volume and swelling of the corrosion shell with further corrosion. The corrosion shell started to peel off from the basalt fiber surface in some areas (State E). In State F, the corrosion shell was partially or entirely peeled off from the fiber core, resulting in a “fresh” surface and reduced diameters *d_1_*. The “fresh” surface of basalt fibers and a reduced diameter *d_1_* lead to an increase in the tensile strength of basalt fibers. On passing State D, the corrosion proceeded with State A again.

### 4.2. Variation Trend of Tensile Strength of Basalt Fibers with Increase of Corrosion Time

As seen from the SEM images (Figure 6 and Figure 7), the corrosion state of basalt fibers immersed in 1 mol/L NaOH solution at 50 °C for 3 days corresponded to State D. The corrosion state of basalt fibers immersed in 4 mol/L NaOH solution at 70 °C for 6 hours corresponded to State F. As confirmed by Figure 3a, the tensile strength increased, corresponding to these corrosion states. Prior to State D, the corrosion state of basalt fibers was in Stage 2. At a temperature of 50 °C, the tensile strength of basalt fibers that has been corroded for one day was lower than the basalt fibers that have been corroded for three days, as shown in Figure 3a. Therefore, it could be concluded that the lowest tensile strength of basalt fibers occurred in Stage 2.

**Figure 12:**
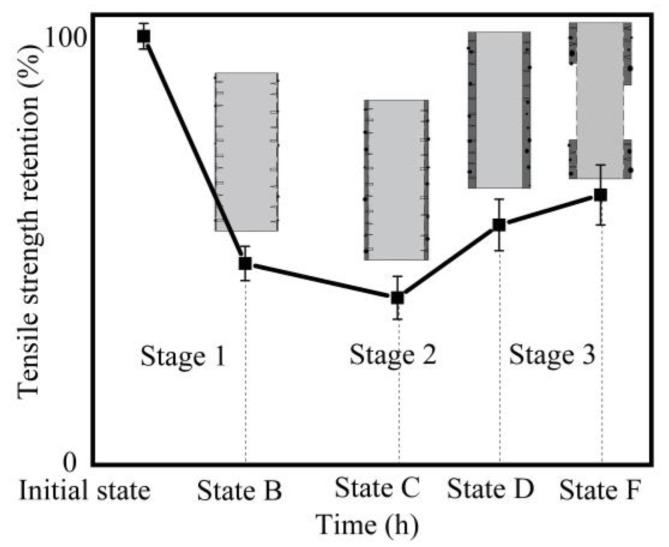
Tensile strength evolution for basalt fibers treated in NaOH solution.

The tensile strength evolution for basalt fibers immersed in NaOH solution was drawn in Figure 12. The corrosion state with the lowest tensile strength was in State C (in Stage 2). On measuring before corrosion State C, it was concluded that the tensile strength decreased consistently as time increased. On measuring before State F, it was found that the tensile strength decreased first and then increased with the increasing time. When the measured corrosion state passed State F, different variation trends were obtained. The formation of a self-peeling corrosion shell was a typical phenomenon in all corrosion conditions [31]. However, the formation time of the corrosion shell was affected by numerous extrinsic and intrinsic factors like fiber component, fiber sizing, solution composition, concentration of solution, temperature and aging time [22,31]. These factors led to different corrosion states of basalt fibers under diverse corrosion conditions; hence, varying trends of tensile strength were observed at different temperatures and concentrations.

Wu et al. [20] conducted that the tensile strength of basalt fibers decreased with increase of time at 25 °C and 55 °C. The main reason was that the used alkaline solution was of very low concentration. Even though the longest corrosion time was 66 days, the SEM images show that a corrosion shell was not formed on the fiber surface. Therefore, the measured corrosion state did not exceed State C, which led to this trend. Ying et al. [21] studied the tensile strength of basalt fiber immersed in 1 and 2 mol/L NaOH solution at 100 °C, respectively. For 2 mol/L NaOH solution, it was found that the tensile strength of basalt fibers decreased initially, then increased and finally decreased. As could be seen from the SEM images, their measured corrosion state has passed State F and had entered State A of the next corrosion process. For 1 mol/L NaOH solution, the tensile strength of basalt fibers decreased first and then increased. It is speculated that a corrosion shell was formed on the fiber surface. This work thus proposed a corrosion mechanism to reveal the changing process of corroded basalt fibers.

### 4.3. Variation Trend of Tensile Strength of Basalt Fibers with Increase of Concentration

The above analysis explained the variation trend of tensile strength of basalt fibers with increase of corrosion time. Figure 3b shows that the variation trends of the tensile strength were similar (decreased first and then increased) at 25 °C and 70 °C. However, the reasons for this phenomenon were different. The increase in concentration of NaOH affects corrosion in two ways: (1) mobility of hydroxyl ions and (2) the rate of reaction. Li et al. [26] studied the alkaline resistance of basalt fibers in 1–5 mol/L NaOH solution at 25 °C. They found that a corrosion shell was not formed on the fiber surface after 2 days of ageing. It could be inferred that the increase in tensile strength of basalt fibers with 6 h ageing was not caused by the corrosion shell. The increase in concentration of NaOH affects corrosion in two ways: (1) mobility of ions and (2) rate of reaction. The increase in concentration led to an acceleration in the reaction-rate for the NaOH solutions. However, the reduced attack at a higher concentration was attributed to a lower mobility of hydroxyl ions.

When the temperature was 25 °C, the increase in concentration increased the amount of hydroxyl ions and thus accelerated the rate of reaction for NaOH solution. However, the mobility of the hydroxyl ions decreased above a certain concentration, causing reduction of the corrosion of basalt fibers [26]. Tensile strength increased at concentration of 4 mol/L. Basalt fibers immersed in high solution concentration corroded more seriously at a high temperature.

When the temperature was 70 °C, the mobility of the hydroxyl ions increased. This caused an increase of corrosion of the basalt fiber, as the concentration increased from 1 to 4 mol/L. However, at a concentration of 4 mol/L, the tensile strength increased due to the peeling off of the corrosion shell (see Figure 7b). In other words, when the temperature was 70 °C, the corrosion state of basalt fibers at a concentration of 4 mol/L passed State C. Therefore, it was observed that the tensile strength first decreased and then increased as the concentration increased from 1 to 4 mol/L. When the temperature was 50 °C, the basalt fibers corroded more seriously with increase of concentration. In addition, all the corrosion states of fibers were prior to State C. Therefore, the tensile strength decreased with increase in concentration (see Figure 3b).

## 5. Conclusions

In this work, the effect of temperature, solution concentration and corrosion time on the corrosion behavior of basalt fibers in alkaline solution was investigated. The main conclusions can be drawn as follows:

1. The hydroxyl ions disrupt the –Si–O–Si– and –Si–O–Al– bonds, leading to the formation of insoluble hydroxides with high calcium and iron content on the fiber surface. With continuation of the hydration reaction, a thin hydrated layer (corrosion shell) covering the whole fiber surface was formed. The corrosion shell caused a significant increase in the strength and elongation at break of basalt fibers.
2. For different temperatures, the degraded fibers showed different variation trends of tensile strength, as the corrosion time increased. When the basalt fibers were immersed in 1 mol/L NaOH solution at temperature of 25 °C, after 1 h, 3 h, 6 h, 1 day and 3 days, their retention ratios of strength were 77.9%, 70.7%, 65.4%, 62.5%, 53.6%, respectively. When the temperature rose to 50 °C, their retention ratios of strength were 67.6%, 57.8%, 52.5%, 49.0%, 58.2%, respectively.
3. A higher temperature accelerated the corrosion rate of basalt fibers in NaOH solution. Due to the leaching of silicon, aluminum and potassium ions, the mass loss ratio of basalt fibers increased with the increase of temperature. For basalt fibers immersed in 1 mol/L NaOH solution for 3 days, the mass loss ratios were 2.4%, 16.6% and 33.8% when the temperature were 25 °C, 50 °C and 70 °C, respectively.

## Figures and Tables

**Table 1:** Quantitative XRF results of desized basalt fibers.

Component Percentage	SiO_2_	Fe_2_O_3_	Al_2_O_3_	CaO	MgO	Na_2_O	TiO_2_	MnO	K_2_O	P_2_O_5_
Desized basalt fibers (wt.%)	47.0	16.0	15.1	9.2	4.1	3.5	1.4	0.2	3.1	0.3

**Table 2:** The tensile properties of the desized basalt fibers.

Parameter	Desized Basalt Fibers
Tensile strength (MPa)	2300 ± 200
Tensile modulus (GPa)	62.6 ± 3
Elongation at break (%)	3.7 ± 0.2

**Table 3:** The comparison of XRF results of the desized and the degraded basalt fibers immersed in 1 mol/L NaOH solution at 70 °C for 3 days.

Component Percentage	SiO_2_	Al_2_O_3_	K_2_O	P_2_O_5_	Fe_2_O_3_	CaO	MgO	TiO_2_	Na_2_O	MnO
Desized basalt fibers (wt.%)	47.0	15.1	3.1	0.3	16.0	9.2	4.1	1.4	3.5	0.2
Degraded basalt fibers (wt.%)	33.9	8.1	2.0	0.1	25.0	15.5	8.8	2.4	3.7	0.5
Percentage change (%)	+13.1	+7.0	+1.1	+0.2	−9.0	−6.3	−4.7	−1.0	−0.2	−0.3

**Table 4:** The element contents of marked areas in Figure 5 and Figure 7.

Measured Point	Element Content (wt.%)										
	O	Na	Mg	Al	Si	P	K	Ca	Ti	Mn	Fe
Point A	38.9	2.7	3.4	9.2	30.6	0.5	2.5	4.9	0.7	0.1	6.6
Point B	42.2	1.8	5.0	5.7	21.4	0.5	1.5	10.0	1.4	0.3	10.4
Point C	49.8	2.8	3.4	7.8	24.8	0.5	1.5	3.8	0.4	0.0	5.2
Point D	50.7	1.8	2.9	2.3	14.2	0.3	0.7	12.6	1.6	0.3	12.5
